# Improving Horticultural Crops via CRISPR/Cas9: Current Successes and Prospects

**DOI:** 10.3390/plants9101360

**Published:** 2020-10-14

**Authors:** Bed Prakash Bhatta, Subas Malla

**Affiliations:** 1 Department of Horticultural Sciences, Texas A&M University, College Station, TX 77843, USA; bhattabp18@tamu.edu; 2 Texas A&M AgriLife Research and Extension Center, Uvalde, TX 78801, USA

**Keywords:** CRISPR, horticulture

## Abstract

Horticultural crops include a diverse array of crops comprising fruits, vegetables, nuts, flowers, aromatic and medicinal plants. They provide nutritional, medicinal, and aesthetic benefits to mankind. However, these crops undergo many biotic (e.g., diseases, pests) and abiotic stresses (e.g., drought, salinity). Conventional breeding strategies to improve traits in crops involve the use of a series of backcrossing and selection for introgression of a beneficial trait into elite germplasm, which is time and resource consuming. Recent new plant breeding tools such as clustered regularly interspaced short palindromic repeats (CRISPR) /CRISPR-associated protein-9 (Cas9) technique have the potential to be rapid, cost-effective, and precise tools for crop improvement. In this review article, we explore the CRISPR/Cas9 technology, its history, classification, general applications, specific uses in horticultural crops, challenges, existing resources, associated regulatory aspects, and the way forward.

## 1. Introduction

The current population of the world is 7.7 billion and is projected to increase to 8.5 billion by 2030, 9.7 billion in 2050, and 10.9 billion by 2100 [1]. The demand for food has gone up and continues to surge with the ever-increasing human population, and as a result, agricultural production has to keep up with the constantly rising demand [2].

However, increased challenges to agricultural production have emerged in recent years, such as the evolution of new races of pests and diseases, increased incidences of drought, heatwaves, changing climates, and other abiotic stresses [3]. Developing high yielding crops that are resistant to biotic and abiotic stresses is one way to address the increasing pressures on agriculture and answer the growing demand for food, feed, and fuel. Conventional breeding and mutation breeding have historically been a successful approach to introduce important genetic variations for crop improvement [4]. However, the diversity of favorable genes or alleles in plants in nature is finite. Additionally, crop improvement via conventional breeding requires extensive time, space, and funding [5]. Transgenic crops have potential as a solution to the limitations of traditional breeding however, the problems associated with them are numerous. These crops are subject to strict regulations regarding their use, import, and export. Ultimately, the potential for positive impact by transgenic foods in global food security is dependent on how the public and governing bodies view the technologies [6]. Currently, there are significant numbers of people advocating against the use of transgenic crops, with some international markets not accepting transgenic crops at all. The availability of transgene-free, genome editing tools using site-directed nucleases (SDN) has opened many paths of opportunity in the field of agriculture. The multifaceted impact of gene-editing tools includes its benefits for human health (e.g., therapeutics, regenerative medicine), and opportunities to improve production qualities and disease resistance of crops and livestock.

Specifically, CRISPR/Cas9 is one of the most recent and widely adopted gene-editing techniques [7]. While it was first reported in the 1980s, the full potential of this method began to be harnessed just a decade ago. During this relatively short period, much interest and debate occurred regarding its use in human, animal, and plant applications. The technique is involved in forward as well as reverse genetics [8]. In humans, particular interest has arisen in managing age-related diseases such as Huntington’s disease [9] and colon cancer [10], and control of heritable diseases such as sickle cell anemia [11]. In animals, several types of research have been carried out, such as increasing body mass in goats [12] and developing avian leukosis virus resistance in chicken [13]. In plants, CRISPR/Cas9 has been extensively used to improve crop disease resistance, which involves knocking-out susceptibility genes and overexpression of resistance genes. Some crops improved for pathogen/disease resistance include powdery mildew resistant wheat [14], cucumber vein yellowing virus-resistant cucumber [15], powdery mildew resistant apple, and grapes [16], blast-resistant rice [17], and canker resistant citrus [18]. However, like every innovation, the technology has been controversial at times and generated public outcry due to the gene-editing of a human embryo by a research group [19].

### 1.1. Reaching the CRISPR Age

Gene or genome editing refers to changes in an organism’s deoxyribonucleic acid (DNA), either by adding, replacing or modifying the genetic material [20]. Gene editing involves the use of SDN through transcription activator-like effector nucleases (TALENs), zinc finger nucleases (ZFNs), and CRISPR/Cas9 [21]. Genome editing tools such as mega nucleases, ZFNs, TALENs, and CRISPR/Cas9 are based on artificially engineered SDN and have been used to introduce mutations through DNA modification in many plant species along with food crops [22]. DNA modifications could be in the form of single-nucleotide polymorphism (SNPs), deletions, insertions, or substitutions. All these gene-editing tools primarily rely on the double-strand breaks (DSBs), which are repaired by the cell’s repair mechanism.

In less than four decades (Table 1), gene-editing technology has undergone tremendous development and transformation. An early example of gene editing is the replacement of the yeast chromosomal segments constructed in vitro by genetic transformation [23]. Similarly, correction of a defective gene having point and deletion mutations were done in mammalian cells using a mutated gene [24].

Several novel tools for either editing or silencing genes evolved in course of time. Mega-nucleases are the oldest SDN based tool used for gene editing. Both ZFNs and ribonucleic acid interference (RNAi) were the groundbreaking technologies developed in the late 20th century, which were used extensively for cutting DNA sequences at specific sites and silencing gene expression. Initial work on RNAi involved an effective interference using double-stranded RNA, which led to the manipulation of gene expression in *Caenorhabditis elegans*, a nematode [42]. Likewise, the earliest research on ZFNs showed that linking of zinc finger proteins with *FokI* endonuclease enables cutting DNA at predetermined sites [43]. Furthermore, TALENs were used to create DSBs at specific, targeted sites [44] and this technique is widely popular as a gene-editing tool in crops.

Many studies have utilized these tools: mega nucleases in maize (*Zea mays*) [45], ZFNs in maize, *Arabidopsis,* and soybean (*Glycine max*) [46,47,48], and TALENs in rice (*Oryza sativa*) [49,50], wheat (*Triticum aestivum*) [14], maize [51], tomato (*Solanum lycopersicum*) [52], and *Arabidopsis* [53]. However, ZFNs and TALENs require complex protein engineering which has limited their broad application in plants [54].

Due to the intensive protein engineering requirement limitations, researchers continued the quest to develop a gene-editing technique to eliminate this challenge. CRISPR/Cas9 technology was first reported as an adaptive immune response in bacteria and archaea to defend against invading viral and plasmid DNA [29,55]. When viruses (bacteriophages) and plasmids infect bacteria, the host chromosome integrates short fragments of phage and plasmid’s nucleic acid as a repetitive element known as CRISPR [56]. Utilizing this nucleic-acid-based immunity stored in its molecular memory, bacteria recognizes new invading phages and plasmids and protect themselves [57]. The main advantage of CRISPR/Cas9 over prior gene-editing techniques is that it does not require complex protein engineering.

### 1.2. CRISPR/Cas9, Novel Variants and Challenges

The recently proposed classification and nomenclature of CRISPR/Cas systems utilize the information obtained from phylogeny and comparative genomic analyses. Based on the associated unique signature Cas proteins (endonucleases or cleaving proteins), there are two major classes of CRISPR/Cas systems. Under Class 1, there are three types of systems: type I, type III, and type IV. These types are more common in archaea than in bacteria [58]. Except for rudimentary Cas-protein for type IV, both type I and type III utilize more than one Cas protein and the effector module is complex. Under Class 2, there are two types of systems: type II and type V. The effector module for Class 2 is comparatively simpler and utilizes a single Cas protein, which is Cas9 the signature protein for type II, which was first discovered as a novel, large protein [59].

**Figure 1:**
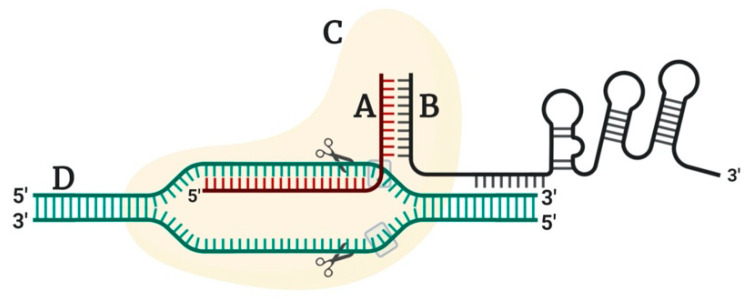
Components of a typical CRISPR/Cas9 construct; A: crRNA; B: tracrRNA; C: Cas9 endonuclease; D: double-stranded target DNA, a grey box with three nucleotides; protospacer adjacent motif (PAM) sequence. Created with BioRender.com.

Among these CRISPR systems, the type II system from *Streptococcus pyogenes* bacteria has been extensively used for gene targeting purposes because of a single, unique effector protein. The CRISPR/Cas9 system utilizes a 20-bp DNA target (guide RNA or gRNA or spacer), followed by a short, trinucleotide (5’-NGG-3’ or 5’-NAG-3’) protospacer adjacent motif (PAM) in the host DNA. The gRNA is duplex in nature and constitutes of CRISPR RNA (crRNA) (homologous to target DNA), and trans-activating crRNA (tracrRNA) [7]. The single gRNA (sgRNA) directs the activity of Cas9 nuclease, thereby creating DSBs and mutations (insertions, deletions, and substitutions) at target-sites [60] (Figure 1). This process of successful DNA-binding and cleavage is only possible when the associated PAM sequence in the host is recognized [61]. The Cas9 endonuclease comprises of two connected lobes: a large globular recognition (REC) lobe and a small nuclease (NUC) lobe [62,63,64]. The REC lobe constitutes of REC1 and REC2 domains, of which REC1 is critical for Cas9 function [64]. The NUC-lobe is a PAM-interacting lobe having two nuclease domains, HNH and RuvC-like, which respectively cleave the DNA strand complementary and non-complementary to the crRNA (target sequence) [7,64,65,66]. The blunt cleavage induced by Cas9 protein are repaired by two major pathways: error-prone non-homologous end joining (NHEJ) or high-fidelity homology-directed repair (HDR) [67]. NHEJ involves the mechanism where DNA ligase IV joins DSBs. During the repair, insertion/deletion of base pairs occurs, leading to a frameshift mutation and/or gene knockout [68]. Contrastingly, HDR involves either an endogenous, natural phenomenon involving sister chromatid as a repair template [63,69] or the introduction of an exogenous repair template DNA (single or double-stranded DNA) in the cut sites to facilitate precise gene editing [67,68,70]. Between the two repair mechanisms, the most common is NHEJ, as it leads to the creation of several mutations in the process of repairing and is a major source of genome rearrangement [69,71]. The difficulties in HDR-mediated DNA repair include competition of the exogenous repair template with the sister chromatid or insufficient delivery of the repair template via *Agrobacterium* or biolistic methods [63].

Derivatives of CRISPR/Cas9 system include base editing that uses dCas9 (dead Cas9), and newly discovered CRISPR/Cpf1 (now CRISPR/Cas12a). dCas9 is also referred to as CRISPR interference (CRISPRi) and it utilizes a modified Cas9 protein. It is very effective in gene silencing by blocking transcription and can serve as an effective tool for targeted gene regulation without disrupting the target sequence [72]. The base editing technology (cytosine-based and adenine-based editors) utilizes dCas9 for the precise editing of a single base without double-strand breaks in the DNA. Using cytosine-based editors, a method has been engineered to convert C (Cytosine) to T (Thymine) and G (Guanine) to A (Adenine) [73]. The adenine-based editor converts A to C and C to G thus completing all four possible transitions [74].

CRISPR/Cpf1 (CRISPR from *Prevotella* and *Francisella* 1) is a single RNA guided system with merits over the CRISPR/Cas9 system, such as a lower rate of off-target edits compared to CRISPR/Cas9 [75,76,77,78]. The T-rich PAM upstream of the target sequence in CRISPR/Cpf1 [79] would potentially increase the number of target regions and help in targeting AT-rich promoter regions [80]. CRISPR/Cpf1 endonuclease can cleave DNA without needing an RNA duplex structure, and the size of its RNA is smaller than the CRISPR/Cas9 guide RNA which makes it simpler and cheaper technology [75]. Using CRISPR/Cpf1 for gene editing makes it possible to insert a new DNA in the target site by the HDR repair pathway, while at the same time introducing random mutations near the target site. This is because Cpf1 cleaves far from the target site allowing multiple rounds of cleavage and repairs [75]. Sticky ends are produced via CRISPR/Cpf1, contrary to blunt ends by Cas9, leading to more precise editing [80].

One of several challenges and concerns associated with CRISPR/Cas9 is the significant problem of off-target effects, which may lead to undesired phenotypic changes in crops [81]. The unpredictable, large on-site deletions created by this technology are also problematic, especially when used in human therapeutics [82]. Another important issue is the ethical concern that can arise with the use of this technology. Important ethical concerns include the potential use of CRISPR as a bioweapon [83] and its application to modify human germ cells/embryos [84] and the potential emergence of alleles overcoming CRISPR gene drives [85].

### 1.3. Applications of CRISPR/Cas9

Of all the available genome editing tools, CRISPR/Cas9 is popular in the plant community. Gene editing is evolving at a rapid pace but CRISPR/Cas9 is still an efficient, precise, and routinely used gene-editing platform. Crops edited with CRISPR/Cas9 have shown high efficiency. These include varying genome efficiencies: for instance, up to 91.6% in rice [86] and up to 79% in maize [87].

CRISPR/Cas9 has the potential to serve as an important plant breeding tool, which has been reflected in the level of interest generated in the plant breeding community. Part of its popularity is due to being simple to design and yet it allows multiplexing to edit multiple loci simultaneously by introducing multiple DSBs [33,88].

Several horticultural crops have been edited using CRISPR/Cas9 technology to meet a diverse array of research objectives including understanding gene function and several applied breeding objectives (Table 2). Some researchers also used CRISPR/Cas9 to lay the foundation for a breeding program by identifying genes contributing to a specific trait. This enables controlled crossing and introgression strategies. Similarly, novel mutations can be introduced directly into elite germplasm, thereby accelerating the breeding program [89].

### 1.4. Existing Resources for CRISPR/Cas9

**Figure 2:**
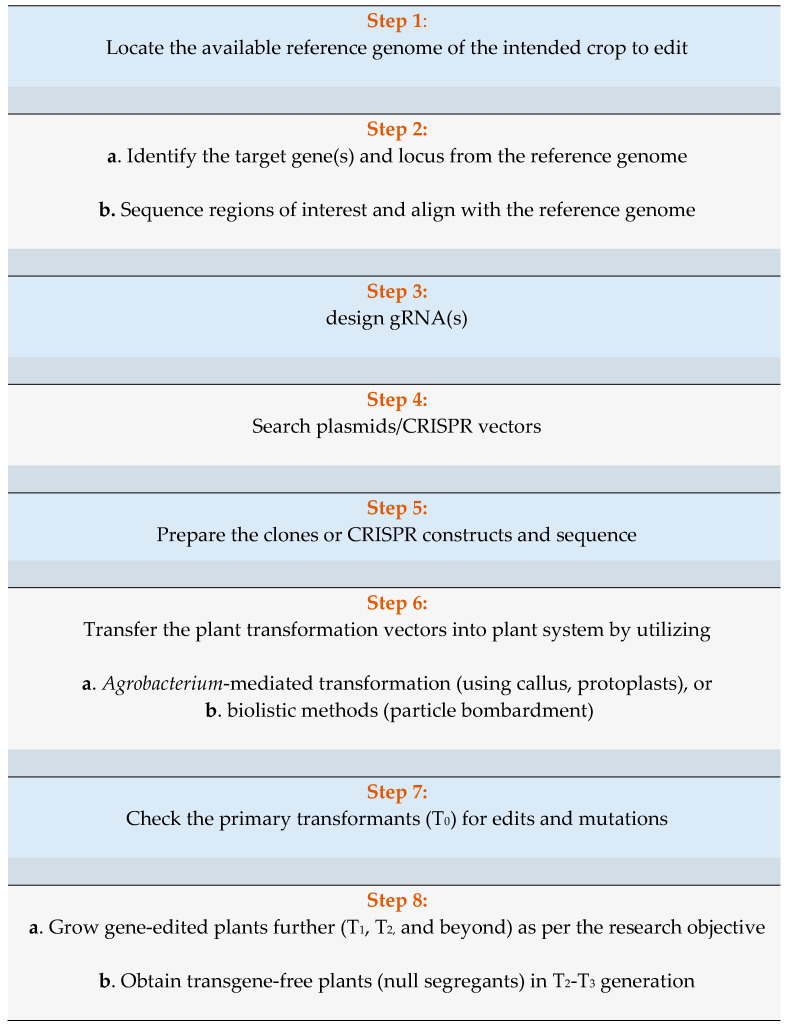
A typical workflow in a CRISPR/Cas9-based gene edit.

Multiple steps are involved in the gene-editing procedure using the CRISPR/Cas9 technique (Figure 2), including designing of gRNAs, the introduction of CRISPR vectors into plant systems, transformation, and analysis of edits in the transformed lines. First, the reference genome for the crop to be edited should be located. Some horticultural crops that have whole genome sequences available are cucurbits (melon, watermelon, cucumber, bottle/wax gourd): http://cucurbitgenomics.org/, solanaceous crops (tomato, potato, pepper, eggplant): https://solgenomics.net/, banana: https://banana-genome-hub.southgreen.fr/, citrus: https://www.citrusgenomedb.org/, apple: https://iris.angers.inra.fr/gddh13/, and spinach: http://www.spinachbase.org/. This is followed by identifying the gene(s) of interest, sequencing them, and ensuring their proper alignment with the reference genome. Next, the gRNAs are designed using several software such as CRISPR-P [138], CRISPR-PLANT [60], CRISPRdirect [139], Chop-Chop [140], and Benchling [141]. After this, a search for CRISPR vectors in plasmid repositories such as Addgene [142] should be completed to assemble the gRNAs and CRISPR/Cas9 cassette. For example, pHSN401, pHSN501, and pHSE401 are used for watermelon transformation [143] and pTC217 is used for tomato transformation [144]. The CRISPR constructs can be prepared using either ligation-dependent [145,146] or ligation-independent protocols [147], and sequenced for proper alignment of the constructs. The steps involved in vector construction can be simulated using software such as Benchling [141] and Snapgene [148].

The transformation vectors are introduced into the plant using either *Agrobacterium*-mediated transformation [145,149,150,151,152,153,154,155,156,157,158] or biolistics [71,159,160]. RNA or DNA viruses have also been used to introduce the gRNA into the plant system [161,162]. Similarly, carbon nanotube nanoparticles have also been used as an efficient plasmid DNA delivery mechanism [163,164]. Researchers have also used protoplasts to first test the cleaving ability of sgRNAs in vivo, thereby determining their efficiency prior to entering into full-scale transformation [165,166]. Primary transformants (T_0_) are checked for edits and mutations via polymerase chain reaction (PCR), Sanger sequencing, restriction digestion, and T7 endonuclease I (T7EI) assay [146,167,168,169,170,171] via software such as TIDE [172] and Snapgene [148] for analysis. The gene-edited plants are advanced one or more generations and confirmed for the absence of transgenic elements via PCR and whole genome sequencing [97,173,174,175]. Specifically, in seed-propagated crops, the T-DNA with the CRISPR/Cas9 construct segregates out in further generations, potentially leading to transgene-free, null segregants. However, in clonally or vegetatively propagated crops, it is challenging to obtain null segregants [176].

### 1.5. The Regulatory Status of Gene-Edited Crops

Multiple crop improvements could be achieved using CRISPR/Cas9 by engineering crops with higher productivity [168,177], improved resistance to diseases [17], resistance to abiotic stresses [178,179,180], and better nutritional quality [94]. This technique has been utilized to create small insertions and deletions that are identical to natural genetic variation [181], which later repair either via the NHEJ pathway or through a donor template-based HDR pathway. Selection against transgenic elements such as the Cas9 and selection markers identifies the null segregants. Whole-genome sequencing can also be done to ensure that there are no traces of CRISPR/Cas9 elements [182] and also to assess the associated off-target effects.

Research in CRISPR/Cas9-based gene editing has focused mostly on NHEJ pathway-based gene editing, as it is a predominant repair pathway in plants [183]. The NHEJ pathway helps create transgene-free plants that do not undergo any regulatory scrutiny [184], as they do not contain foreign elements present in GMOs, and the mutations induced are similar to what would occur naturally as random mutagenesis. Therefore, plants generated from NHEJ-based editing are not currently regulated once null segregants are obtained. NHEJ enables rapid crop breeding (less than five years) [185,186] as compared to traditional breeding where it typically takes more than 10 years to develop a variety [187]. Similarly, in HDR, exogenously provided homologous DNA sequences (template) are used to precisely repair the DSBs in DNA [188]. Gene addition using HDR, to some extent, may deem it transgenic [189]. However, if the repair template is cisgenic, where genes from the same species, related interbreeding, or wild species are used, the resultant crops may not be considered transgenic. Nevertheless, the regulatory framework regarding NHEJ and HDR-mediated gene editing is perceived and defined in different respective contexts depending on the country. 

In the United States, the United States Department of Agriculture (USDA), Food and Drug Administration (FDA), and the Environmental Protection Agency (EPA) are the agencies that oversee the regulation of genetically modified organisms (GMOs) [190]. Due to the removal of transgenic elements in plants, regulatory agencies in the USA do not consider CRISPR-edited plants as GMOs for regulatory purposes. CRISPR-edited plants can be deployed widely in less time and at a lower cost than conventional plant breeding [191].

In 2016, regulatory approval was provided by USDA to a waxy corn null segregant line [192], as it was not a plant pest and did not contain transgenes owing to selection in subsequent segregations. In the same year, the USDA stated that the CRISPR/Cas9 edited, anti-browning mushrooms need not be regulated as they contain no foreign DNA integrated to the mushroom genome [193]. Lines approved by regulatory agencies can be directly tested in the field, benefitting researchers and biotechnology companies [194], and help in the development of new cultivars in a limited timeframe with reduced costs.

In the USA, genome editing has been allowed as a potential expansion of traditional plant breeding tools for crop improvement [195]. A catalog of regulatory inquiries and approvals for gene-edited crops in the USA is available [196]. Several letters of inquiries have been received for gene-edited crops/organisms, including tomato, citrus, pennycress, soybean, sugarcane, camelina, petunia, flax, rice, and orchid—all of which received the regulatory waiver. Similarly, in May 2020, an updated biotechnology framework has been developed by USDA-APHIS and has been defined as SECURE (Sustainable, Ecological, Consistent, Uniform, Responsible, Efficient) rule [197]. The framework provides three exemptions to make a single genetic modification to any plant species: (i) changes resulting after DSB in DNA in absence of an external repair template, (ii) targeted single base pair substitution, and (iii) introduction of a known gene that exists in the plant’s gene pool [198].

The regulation of gene-edited crops differs based on the legal framework in each nation or group of nations. In Canada, only if the gene-edited product (plant, feed, or food) is novel, i.e., different from what is already available, it undergoes the pre-market assessment [199]. In Europe, there are stringent regulations on CRISPR/Cas9 [200]. Recently, the Court of Justice of the European Union, the highest court of Europe, ruled that gene-edited crops be treated with the same set of regulations that are imposed on genetically modified (GM) organisms [201]. In contrast, Australia has taken the middle ground, allowing the use of gene-editing without introducing any foreign genetic material [202]. There is also a favorable environment for gene-edited crops in Asian countries such as China and Japan, where several field trials of gene-edited crops have been reported [203]. In India, there is a regulation mechanism for genetic engineering which includes “modification of an organism or in a cell by deletion and removal of parts of the heritable material” [204]. In South Africa, GMO regulations are in place but there is still a need for genome-editing-specific legislation [205]. In Argentina, the regulatory framework is on a case-by-case basis taking into consideration the process involved, i.e., the breeding methodology used, new trait or characteristic introduced, and evidence of the genetic changes present in the final product [206].

### 1.6. Way Forward

There is immense potential for gene editing techniques including novel plant breeding tools such as CRISPR/Cas9 in enhancing production, productivity, quality, and nutritional characteristics of horticultural crops. This technology is expected to contribute to resolving the food deficit issues prevailing in the world. Acceptance of plant innovation and a congenial regulatory atmosphere in the USA allows gene-edited plant products with the market potential to be extensively researched and appear on the grocery shelf [207]. This includes TALEN-edited Calyno^TM^ high-oleic soybean oil [208]. The primary concern currently is producer and consumer acceptance to the CRISPR-edited products commercially available [209,210]. Proponents of gene editing argue that characteristics of the final product, not the process involved, should be considered for food safety assessment [211]. However, there will still be differing perceptions of gene-edited products, specifically due to the varying regulatory provisions in different countries. A global scientific consensus and uniform regulatory measures across countries might add to the usefulness of gene-editing technology beyond the domain of research.

## Figures and Tables

**Table 1:** Timeline of events in clustered regularly interspaced short palindromic repeats (CRISPR) discovery and use.

1987–1995 Short direct repeats observed in *Escherichia coli*, *Haloferax mediterranei*, and *Haloferax volcanii* [25,26,27]
2002 The term “CRISPR” coined, CRISPR components identified/named [28]
2005 CRISPR speculated as a defense mechanism in bacteria [29]
2007–2008 CRISPR/Cas genes confirmed to provide resistance to phages/ explanation of antiviral defense mechanism [30,31]
2010 CRISPR/Cas system can specifically cleave double-strand DNA [32]
2012 Cas9 endonuclease guided by RNA for gene editing [7]
2013 Human genome-edited by CRISPR/Cas9 system [33,34] First use of CRISPR/Cas9 in plants [14,35,36,37]
2014– Routine application of CRISPR/Cas9 for crop improvement [38,39,40,41]

**Table 2:** Examples of horticultural crops where CRISPR/Cas9 technology was used to meet research objectives.

Crop.	Research Objective Met Using CRISPR/Cas9	References
Tomato	Understand the role of a photoreceptor in seedling development/stress tolerance	[90]
	Bacterial speck resistance	[91]
	Combine desired traits with useful traits present in wild type	[92]
	Confirm function of a gene involved in Fusarium wilt tolerance	[93]
	Improve lycopene content	[94]
	Develop Tomato Yellow Leaf Curl Virus resistance	[95]
	Long shelf life, generate parthenocarpy	[96]
	Transgene-free powdery mildew resistant plants	[97]
	Achieve ideotype	[98]
	Develop day-neutral and early yielding plants	[99]
Capsicum	Understand the role of a transcription factor in chloroplast development and fruit color	[100]
Carrot	Generate haploid plants	[101]
Potato	Reduce enzymatic browning	[102]
	Overcome self-incompatibility	[103,104]
	Reduce steroidal glycoalkaloids	[105]
	Develop amylopectin starch cultivars	[106]
Sweet potato	Enhance Fusarium wilt resistance	[107]
Watermelon	Validate function of vacuolar sugar transporter gene	[108]
	Obtain gynoecious genotypes	[109]
	Resistance to *Fusarium oxysporum* f. sp. *niveum* Race 1	[110]
	Functional characterization of a gene in fruit flesh sugar accumulation	[111]
	Herbicide resistance	[112]
Pumpkin	Understanding the role of root apex in salt tolerance	[113]
Cucumber	Transgene-free gynoecious plants	[114]
	Broad virus resistance	[15]
Cabbage	Compare delivery methods in model genes	[115]
	Target flowering-time regulator gene	[116]
	Generate early flowering phenotype	[117]
	Multiplex gene editing to overcome self-incompatibility and produce male-sterile lines	[118]
Lettuce	Generate seedlings capable of germinating at higher temperatures	[119]
Cassava	Brown streak resistance	[120]
Strawberry	Characterize a transcription factor involved in anther development	[121]
	Identify genes involved in auxin accumulation and biosynthesis	[122]
Citrus	Canker resistance	[18,123,124]
Apple	Reduce fire blight susceptibility	[125]
Banana	Inactivate banana streak virus	[126]
	Basis of generating dwarf and semi-dwarf cultivars	[127]
Grapes	Study editing efficiency	[128]
	Obtain biallelic mutations in the first generation	[129]
Papaya	Study the evolution of oomycetes in evading plant defense mechanism	[130]
Cacao	Edit gene involved in suppressing defense response	[131]
Coffee	Proof-of-concept to knock out genes of interest	[132]
Petunia	Understand genes involved in flower longevity and ethylene production	[133]
Orchid	Understand the MADS gene family expressed in floral organs	[134]
Chrysanthemum	First report of gene editing	[135]
Japanese morning glory	Flower longevity	[136]
	Understand the role of a gene in petal coloration	[137]
